# Time-of-day effects on muscle mitochondria following short-term ablation of satellite cells

**DOI:** 10.3389/fphys.2025.1613184

**Published:** 2025-07-02

**Authors:** Ryan E. Kahn, Fawzan Dinnunhan, Guadalupe Meza, Richard L. Lieber, Orly Lacham-Kaplan, John A. Hawley, Sudarshan Dayanidhi

**Affiliations:** ^1^ Exercise and Nutrition Research Program, The Mary MacKillop Institute for Health Research, Australian Catholic University, Melbourne, VIC, Australia; ^2^ Shirley Ryan AbilityLab, Chicago, IL, United States; ^3^ Feinberg School of Medicine, Northwestern University, Chicago, IL, United States; ^4^ Hines VA Medical Center, Maywood, IL, United States; ^5^ Department of Sport and Exercise Sciences, Institute of Sport, Manchester Metropolitan University, Manchester, United Kingdom

**Keywords:** muscle fatigue, muscle mitochondria, contractility, molecular clocks, satellite cells

## Abstract

**Introduction:**

Endurance exercise capacity fluctuates by time-of-day due, in part, to molecular clock effects on muscle physiology. As endurance-based exercise relies predominantly on mitochondria for the conversion of cellular energy, fluctuations observed in endurance capacity have been attributed to diurnal variation in mitochondrial respiration and molecular clock KO animals exhibiting blunted mitochondrial function/content. Recently, a circadian profiling of satellite cells (SCs) demonstrated molecular clock, metabolic, and mitochondrial genes exhibit robust oscillation over 24 h while long-term SC ablation impairs endurance exercise capacity. These lines of evidence suggest SC molecular clocks may influence mitochondrial respiration according to time-of-day. We determined whether mitochondrial respiration differs by time-of-day in the presence and absence of SCs in oxidative (soleus, SOL) and glycolytic (tibialis anterior, TA) muscle.

**Methods:**

Utilizing a Pax7^CRE−ERT2/+;^ Rosa26^DTA/+^ mouse model capable of SC ablation (SC^+^, SC^−^), we conducted experiments in either the morning, afternoon, or evening.

**Results:**

In both SOL and TA, respiratory coupling ratio (RCR) was lowest and Leak-state respiration (TA) was highest in the morning with no differences observed following SC ablation. Utilizing a submaximal ex vivo fatigue protocol that relies predominantly on mitochondrial energy, we observed that submaximal fatiguability was lower in the morning than afternoon in glycolytic muscle (EDL) (morning-SC^**+**^: 54 ± 5; afternoon-SC^**+**^: 36 ± 6 contractions until fatigue, *p* < 0.05), which corresponded with peak/trough Bmal1 and *CLOCK* gene expression in muscle.

**Discussion:**

Collectively, the results from the current study suggest that SCs influence mitochondria in a time-of-day manner.

## Introduction

In situations where skeletal muscle mitochondria are the primary source of cellular energy to sustain contractile demands, endurance exercise capacity is a function of the time of day (Maier et al., 2021; Adamovich et al., 2021; Ezagouri et al., 2019; Jordan et al., 2017). Muscle mitochondria also exhibit time-of-day rhythmicity in *ex vivo* maximal respiration (Gemmink et al., 2023; van Moorsel et al., 2016), due, in part, to oscillatory regulation by molecular clocks, whereas knock-out (KO) mouse models of molecular clock gene *Bmal1* have decreased muscle mitochondrial content and respiration (Andrews et al., 2010), highlighting the important role of molecular clocks in mitochondrial function.

Skeletal muscle repair after exercise-induced muscle damage is primarily mediated by satellite cells (SCs), the resident muscle stem cell population (Snijders et al., 2015; Mackey and Kjaer, 2017). SCs rhythmically express clock, contractile, and mitochondria-related genes on a diurnal basis (Solanas et al., 2017), suggesting that SCs may regulate contractile and mitochondrial function and, consequently, endurance exercise capacity—according to the time of day. Recently, we demonstrated that maximal isometric and eccentric contractile functions were altered in the presence/absence of SCs in the morning versus afternoon (Kahn et al., 2024), suggesting that the SC molecular clock (Solanas et al., 2017) exerts a time-of-day effect on muscle contractile characteristics. Furthermore, we have reported that inducible depletion of *Bmal1* within SCs alters *in vivo* and *ex vivo* force production, which leads to concordant alterations in damage/repair following contractile injury; these findings highlight a direct effect of SC molecular clocks on contractile function (Kahn et al., 2025).

Following SC ablation, submaximal endurance exercise capacity is altered (Englund et al., 2020; Jackson et al., 2015), with such perturbations unaccompanied by changes in mitochondrial content, succinate dehydrogenase (Complex II, CII), or muscle size. Although mitochondrial function directly impacts exercise capacity, mitochondrial respiration was not directly measured in that study (Jackson et al., 2015). Whether mitochondrial respiration is altered in the presence or absence of SCs and whether this relationship is time-of-day-dependent remain unknown. Additionally, mitochondrial respiration differs based on the oxidative capacity of the tissue, with highly oxidative muscle (e.g., the soleus, SOL) exhibiting much greater respiration than glycolytic muscle (i.e., the tibialis anterior, TA). Accordingly, the purpose of this study was to determine the time-of-day differences in mitochondrial respiration in both glycolytic and oxidative muscles—measured in the morning, afternoon, and evening—in the presence or absence of SCs. We hypothesized that variations in time-of-day mitochondrial respiration would depend on the presence of SCs. We also tested whether *ex vivo* submaximal fatigue was a function of time of day.

## Methods

### Animal information

Pax7^CRE−ERT2/+;^ Rosa26^DTA/+^ mice (n = 43; male mice = 20; female mice = 23; 4–10 months of age) were used for these experiments (Murach et al., 2021; Englund et al., 2021). All animal experiments were performed with the approval of the Northwestern University Institutional Animal Care and Use Committee (protocol number: IS000019422). Mice were euthanized via CO_2_ inhalation followed by cervical dislocation. Mice were housed in groups of 2–5 animals per cage in a temperature/humidity-controlled environment on a 14:10 light–dark cycle (lights on at 06:00 h) and were provided ad libitum access to food/water. Prior to all mitochondrial respiration experiments, animals underwent an 8–12-h fast to avoid any confounding effects of cellular energy status on mitochondrial respiration. Utilizing the Cre-Lox system, inducible depletion of SCs was achieved via five consecutive days of oral gavage with tamoxifen, while control (vehicle) animals received oral gavage with peanut oil (2 mg in 100 μL per day) (Relaix and Zammit, 2012; Fry et al., 2015), followed by a minimum 14-day washout period (26 ± 1 day). SC ablation using this model is similar across hindlimb muscles (McCarthy et al., 2011). Animals were randomly assigned a treatment group (SC^−^–tamoxifen or SC^+^–vehicle) and experimental timepoint [morning—07:00 h (ZT1), afternoon—15:00 h (ZT9), and evening—19:00 h (ZT13); lights on at 06:00 h (ZT0)]. All groups had a mixture of both sexes. All experiments were performed immediately after euthanasia at the assigned timepoints. Animals were euthanized via CO_2_ inhalation at a flow rate of 8 L/min.

### Muscle mitochondrial respirometry

High-resolution mitochondrial respirometry was performed using the Oroboros O2k system (Oroboros Instruments, Innsbruck, Austria), following published protocols (Guo et al., 2022; Thompson et al., 2023). Immediately following euthanasia, the soleus and TA were dissected and placed in an ice-cold BIOPS solution (2.77 mM CaK_2_EGTA, 7.23 mM K_2_EGTA, 5.7 mM Na_2_ATP, 6.56 mM MgCl_2_, 20 mM taurine, 15 mM Na_2_-phosphocreatine, 20 mM imidazole, 0.5 mM DTT, and 50 mM MES). The muscles were then further dissected under a microscope and mechanically separated in ice-cold BIOPS to obtain two replicates of 2–3 mg from each muscle. Following mechanical separation, muscles were chemically permeabilized with 50 μg/mL saponin for 30 min at 4°C and were subsequently washed for 10 min in mitochondrial respiration media [MiR05; 0.5 mM EGTA, 3 mM MgCl_2_, 60 mM K-lactobionate, 20 mM taurine, 10 mM KH_2_PO_4_, 20 mM 4-(2-hydroxyethyl)-1-piperazineethanesulfonic acid, 110 mM sucrose, and 1 g/L fatty acid-free bovine serum albumin]. Respirometry experiments on both samples from TA and soleus were run simultaneously and conducted at 37°C under hyperoxygenated (200–450 μM O_2_) conditions in MiR05 to avoid any limitations/differences related to oxygen diffusion.

To assess maximal phosphorylation and electron transport chain (ETC) capacity of complex I (CI)- and CII-mediated respiration, we used a sequential substrate–uncoupler–inhibitor titration (SUIT) respiration protocol. Specifically, we measured (final concentrations in the O2k chamber are indicated in parentheses) 1) LEAK respiration (L) after TCA cycle stimulation with NADH-linked substrates such as pyruvate (5 mM) and malate (2 mM) to support electron flow through CI of the ETC; 2) CI-supported respiration (OXPHOS, P_CI(D)_; “State 3” respiration) after the addition of adenosine diphosphate (ADP, 5 mM); 3) maximum CI-supported respiration (OXPHOS; P_CI_) after the addition of glutamate (10 mM); 4) maximum CI- and CII-supported respiration (OXPHOS; P_CI+II_) after the addition of CII substrate succinate (10 mM); 5) uncoupled respiration, representing maximum ETC capacity (E_CI+II_), after step-wise titration of the uncoupler carbonyl cyanide m-chlorophenyl hydrazine (CCCP; 0.5 mM); 6) maximum ETS capacity in the presence of CII substrate only (E_CII_) after the addition of the CI-inhibitor rotenone (0.5 mM); 7) residual, non-mitochondrial oxygen consumption (ROX) after inhibiting complex III (CIII) of the ETC with antimycin A (2.5 mM). Prior to the administration of succinate, cytochrome-c was administered to assess for over-permeabilization of the mitochondrial membrane. Any value higher than 15% following cytochrome-c administration was considered over-permeabilized, and these data were not used. The respiratory control ratio (RCR) was calculated as the ratio of State 3 [P_CI(D)_] to LEAK respiration. For all respirometry experiments, animals underwent an 8–12-h fast to account for potential differences in diurnal energy status or variation in food intake.

### Mitochondrial activity assays

Immediately after euthanasia, the TA from the contralateral limb was flash-frozen in liquid nitrogen-cooled isopentane and stored at −80°C. Muscles were powdered using a liquid nitrogen-cooled mortar and pestle and then homogenized in 0.5 mL of Zheng buffer (210 mmol/L mannitol, 70 mmol/L sucrose, 5 mmol/L 4-[2-hydroxyethyl]-1- piperazineethanesulfonic acid [HEPES], and 1 mmol/L ethylene glycol-bis(b-aminoethyl ether)-N,N,N′,N′- tetraacetic acid [EGTA] [pH 7.2]) (Dayanidhi et al., 2021; LaBarge et al., 2016). Homogenization was performed on ice using glass-on-glass conical tissue grinders undergoing 16 strokes at 500 rpm. Homogenized tissue was subsequently centrifuged at 600 *g* at 4°C for 10 min. Supernatant protein concentration was ascertained using the Pierce™ Bicinchoninic Acid Protein Assay Kit according to the manufacturer’s instructions (Thermo Fisher Scientific, Waltham, MA, United States) (Dayanidhi et al., 2021). Based on protein concentrations, aliquots of equal protein concentrations were made to perform citrate synthase (CS) and electron transport chain complex-I (ETC-C-I) enzymatic activity assays.

For the CS assay, which enzymatically assesses the TCA cycle reaction mediating the conversion of acetyl coenzyme A (acetyl-CoA) to citrate, Tris buffer (pH 8.0) was used with 12.5 mmol/L acetyl coenzyme A. To catalyze the reaction from acetyl-CoA to citrate via citrate synthase, 5 mmol/L oxaloacetic acid was administered to initiate the actions of citrate synthase, and the subsequent absorbance rate of the reactionary byproduct 5,50-dithiobis–(2-nitrobenzoic acid) was measured every 15 s for 3 min to determine the rate of citrate synthase activity (Dayanidhi et al., 2021; Leckey et al., 2018). Assays were performed on 10 μg of protein per well, in triplicate, in a 96 well-plate at a wavelength of 412 nm.

To assess ETC complex-I activity, 2 mmol/L of NADH was used as a substrate to be oxidized by CI, and 5 mmol/L of ubiquinone (coenzyme-Q) was administered to facilitate the passing of electrons from CI to CIII. The rate of reduction in absorbance in NADH fluorescence over 3 min was used as a surrogate for the rate of CI’s oxidation of NADH to NAD^+^ and, thus, its maximal electron flux capacity. To assess the test’s specificity for CI, 1 mmol/L of rotenone was administered. All assays used 30 ug of protein per well, in triplicate, in a 96 well-plate at a wavelength of 340 nm.

The mitochondrial copy number was determined as previously described (Dayanidhi et al., 2021). In brief, following DNA purification, DNA concentration and purity were assessed spectrophotometrically. We used primers for a mitochondrial gene (Cox1) and a nuclear gene (ribosomal L13a) (Integrated DNA Technologies, Coralville, IL) with Terra qPCR Direct TB Green (TaKaRa Bio Inc., Mountain View, CA, United States) to perform real-time PCR to measure the mitochondrial DNA (mtDNA)-to-genomic DNA ratio. The forward and reverse primers of mitochondrial and nuclear genes used in this assay were as follows: mtDNA COX1–F 5′-AGA TGT AGA CAC CCG AGC CT-3′, mtDNA COX1–R 5′-GGC TCA TAA TAT GGC GGG GG-3’; ribosomal L13a–F 5′-TGC TCA CAG ACT CTC AGG-3′, and ribosomal L13a–R 5′-AAG CCT TCC TCT TTC CAC AGG-3’. To calculate the mitochondrial copy number, Pfaffl’s model was used to assess the difference in the threshold cycle between mitochondrial/nuclear gene pairs and expressed in arbitrary units (Pfaffl, 2001).

### Gene expression

Immediately after euthanasia, the quadriceps muscle was harvested and snap-frozen in liquid nitrogen-cooled isopentane for gene expression assays. Genes of interest were analyzed using the QX200 AutoDG Droplet Digital PCR system (Bio-Rad). Extracted RNA was analyzed for quality using NanoDrop 2000 and quantified using Qubit. Following this, an absorbance ratio of <1.8 was used to assess RNA quality, and quantification was performed to equilibrate the samples to 1 ng/μL prior to ddPCR. A total of 5 μL of the equilibrated sample was aliquoted into the 96-well ddPCR plate along with 17 μL of a master mix (One-Step RT-ddPCR advanced kit for probes). The master mix also consisted of florescence-labeled ddPCR primers (Bio-Rad) for the housekeeping gene glyceraldehyde 3-phosphate dehydrogenase (*GAPDH*) and the gene of interest. Molecular clock genes of interest were *Bmal1 (brain and muscle ARNT-like protein 1), CLOCK, Cry1 (gene cryptochrome 1),* and *Per2 (period circadian regulator 2)*; mitochondrial dynamics/morphology genes of interest were *Opa1* and *Fis1 (fission, mitochondrial 1)*; metabolic genes of interest were *Pdk4 (pyruvate dehydrogenase kinase 4)* and *PCG1a (peroxisome proliferator-activated receptor gamma coactivator 1-alpha).* The plate was then placed into the AutoDG to generate up to 20,000 droplets per well, followed by reverse transcription. The plate was read using the QX200 Droplet Reader to visualize and yield exact gene concentration per μL of sample and was subsequently expressed relative to *GAPDH* concentration per well. For SC^+^ and SC^−^ groups, all genes of interest across timepoints were normalized to the same respective gene (represented as fold-change) for either morning-SC^+^ or morning-SC^−^ groups. Time-of-day changes observed in molecular clock gene expression served as a guide for muscle physiology experimental timepoints as prior work has shown that variation in muscle function is associated with time-of-day differences in clock gene expression (Douglas et al., 2021; Gutierrez-Monreal et al., 2020; Adamovich et al., 2021; Ezagouri et al., 2019).

### *Ex vivo* contractile experiments

Immediately after euthanasia, the extensor digitorum longus (EDL) was isolated by tying 5.0 silk sutures to the proximal/distal tendons while immersed in Ringer’s solution (in mM: 137 NaCl, 5 KCl, 1 NaH_2_PO_4_, 24 NaHCO_3_, 2 CaCl_2_, 1 MgSO_4_, and 11 glucose), containing 10 mg/L curare, with a pH of 7.5. For *ex vivo* contractile measurements, the EDL was mounted between a force transducer (Aurora Scientific 300C) and a motor in a custom bath filled with oxygenated Ringer’s solution at 37°C, with platinum electrodes straddling the muscle as previously described (Palmisano et al., 2015; Chapman et al., 2014; Sam et al., 2000).

Muscle stimulation protocols were based on prior work (Palmisano et al., 2015; Chapman et al., 2014; Sam et al., 2000). In brief, after the EDL was mounted and allowed to stabilize for 5 min, optimal muscle length (L_o_) and current were determined through a series of twitch contractions. Force and length from each contraction were acquired using a custom LabVIEW program. The raw contractile force was converted to Newtons (N) and expressed as specific force (N/cm^2^) by normalizing to EDL physiological cross-sectional area (PCSA) (Palmisano et al., 2015; Chapman et al., 2014; Sam et al., 2000).

A force–frequency curve (300 m per contraction; 3 min rest between contractions; 10, 20, 30, 40, 50, 60, 70, 100, and 110 Hz) was constructed to identify maximal tetanic force (P_o_). Subsequently, the frequency corresponding to ∼50% P_o_ (∼25 Hz) was used for 300 m trains of submaximal fatiguing contractions at intervals similar to previous reports (Nogueira et al., 2022; Gandra et al., 2012; Nogueira et al., 2011). The fatigue protocol started at 50% P_o_ and ended at 15% P_o_. The first 10 contractions were separated by 10 s each, followed by the next 10 contractions separated by 7 s, with all remaining contractions administered at 5-s intervals until cessation (15% P_o_). The number of contractions needed to reach 15% P_o_ from 50% P_o_ was defined as the fatigue index.

We validated that this submaximal fatigue protocol relied predominantly on mitochondria for contractile-energetic needs by inhibiting mitochondria (Delfinis et al., 2022). In a subset of mice (n = 3), immediately after completion of the force–frequency contractions, Ringer’s solution was replaced with Ringer’s containing 10 mM oligomycin (an inhibitor of mitochondrial ATP synthase). The muscle was allowed to remain in this solution for 10 min (Delfinis et al., 2022). Maximal force and submaximal fatigue were compared between only Ringer’s and Ringer’s with oligomycin. Following this validation in a subset of mice, we then moved forward with performing submaximal fatigue experiments on the EDL of morning-SC^**+**^ and afternoon-SC^**+**^ animals.

### Immunohistochemistry

Gastrocnemius muscle was flash-frozen in liquid nitrogen-cooled isopentane, stored at −80°C, and transferred to a −25°C cryostat for sectioning and subsequent immunohistochemical staining. In brief, the muscle was allowed to equilibrate in the cryostat for 1 h before sectioning, embedded in a cryomold, flash-frozen in liquid nitrogen-cooled isopentane for 10 s, and then re-equilibrated in the cryostat for 30 min (Meyer et al., 2022). Muscle sections were cut at 10 μm, and following sectioning, slides were allowed to air-dry for 1 h and were subsequently stored at −80°C. In brief, after thawing, slides were fixed in 4% PFA, incubated in 3% H_2_O_2_ to block endogenous peroxidases, and washed with PBS. A heat-induced epitope-retrieval step (slides steamed for 30 min in a pressure cooker in a citrate buffer bath) was performed, and the slides were permeabilized in 1% Triton-X thereafter. Following this, the slides were blocked for 1 h in 1% BSA mouse-on-mouse blocking buffer and incubated in primary antibody solution overnight. The following day, the slides were incubated for 90 min in a biotinylated secondary antibody, washed, and incubated in the secondary antibody solution for 1 h. The slides were then incubated in an amplification solution for 20 min, washed, mounted using VECTASHIELD with DAPI, and cover-slipped (Wen et al., 2018; Dungan et al., 2019). Primary antibodies used were anti-Pax7 [mouse IgG1, 1:100 DSHB, concentrate] and anti-laminin (rabbit IgG, 1:500, Sigma-Aldrich, L9393]. Secondary antibodies for Pax7 were goat anti-mouse IgG1 biotin-SP-conjugated (1:1,000, Jackson ImmunoResearch, 115-065-205), streptavidin-horseradish peroxidase (SA-HRP) (1:500, Invitrogen, S-911), and SuperBoost Tyramide reagent Alexa Fluor 594 (1:500, Thermo Fisher, B40957), while for laminin, Alexa Fluor 488 goat anti-rabbit IgG (H + L) (1:250, Invitrogen, A-11034) was used. The slides were imaged at ×20, and entire cross-section images were analyzed for myofiber cross-sectional area (CSA) and SC abundance. Cross-sectional area and SC quantification were carried out via blinded–automated analyses using MuscleJ (Mayeuf-Louchart et al., 2018).

### Statistical analysis

Comparisons of mitochondrial respiration states, mitochondrial activity, and gene expression were analyzed using two-way ANOVA to assess the main effects of time of day and SC ablation, followed by Tukey’s post hoc comparisons. Comparisons of *ex vivo* contractility between morning and afternoon groups were analyzed using unpaired t-tests. All immunohistochemistry (IHC) data were analyzed using unpaired t-tests. Specific statistical tests are indicated in figure legends. All statistical analyses were performed using Prism 9.0 (GraphPad, San Diego, CA). All data in the results are reported as the mean ± standard error of mean (SEM).

## Results

### Satellite cell ablation and myofiber area

**FIGURE 1:**
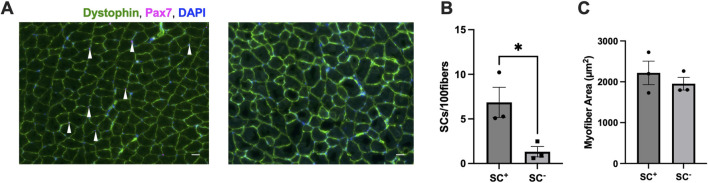
Satellite cell (SC) ablation and myofiber area. **(A)** Representative images of muscle cross-sections labeled for laminin and Pax7 showing the presence of SCs and their absence (right) (scale bar set to 100 µm). **(B)** Ablation of Pax7^+^ SCs following tamoxifen administration. **(C)** Myofiber area in SC^**+**^ versus SC^**−**^ animals (µm^2^). All IHC data were analyzed on the gastrocnemius and auto-quantified using MuscleJ. All data are shown as the mean ± s.e.m. All groups were analyzed using unpaired t-tests (*p < 0.05) (n = 3 per group).

Tamoxifen treatment induced an ∼80% reduction in SCs [Pax7^+^/DAPI + cells (SC^**+**^: 7 ± 2; SC^**−**^: 1 ± 2 SCs/100 fibers, *p* < 0.05] (Figures 1A,B). The average myofiber area was not different between SC^**+**^ and SC^**−**^ groups (SC^**+**^: 1954 ± 155; SC^**−**^: 2,218 ± 288 μm^2^) (average number of fibers used for calculations: SC^**+**^: 2,693 ± 185; SC^**−**^: 3,424 ± 918) (*p* > 0.05) (Figure 1C).

### Mitochondrial respiration in the soleus across the time of day in the presence and absence of SCs

**FIGURE 2:**
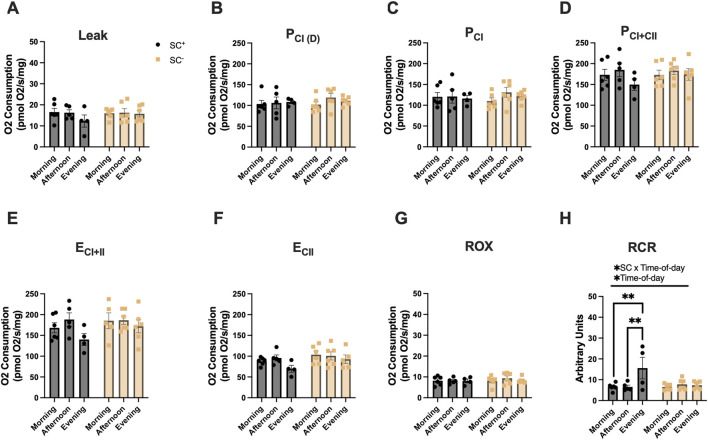
Mitochondrial respiration in the soleus across different times of day in the presence and absence of SCs. **(A–H)** Mitochondrial respiration across all states and in the presence/absence of SCs in the SOL. All groups were compared using two-way ANOVA for main effects of time-of-day and SC status, followed by Tukey’s post hoc comparisons (n = 4–7 per group) (**p < 0.01). (Leak: PM, P_CI(D): D, P_CI: G, P_CI + II:S, E_CI + II: CCCP, E_CI: R, and ROX: Ama). [P, pyruvate; M, malate; Oct, octanoylcarnitine; D, adenosine diphosphate (ADP); G, glutamate; S, succinate; R, rotenone (inhibitor of complex I); Ama, antimycin A (inhibitor of complex III)].

We first assessed whether mitochondrial capacity differed between glycolytic and oxidative muscles. Overall, maximal OXPHOS capacity (P_CI+II_) of the soleus was greater than that of the TA, regardless of treatment (soleus-SC^**+**^: 170 ± 9, TA-SC^**+**^: 118 ± 7, soleus-SC^**-**^: 178 ± 6, and TA-SC^**-**^: 117 ± 5 pmolO_2_/s/mg) (p < 0.05). In the SOL (Figures 2A–H), an overall interaction effect between SCs and time of day, along with an effect of time of day, was observed for RCR; Tukey’s post hoc comparisons showed that the RCR was highest in the evening across all timepoints in SC^+^ groups, while no such time-of-day differences were observed in SC^−^ groups (*p* < 0.01 and *p* < 0.05, respectively) (Figure 2H; Table 1).

**TABLE 1:** All mitochondrial respiration data (T1) and mitochondrial oxygen consumption values across all states, muscles, groups, and timepoints (pmol O_2_/s/mg). All data are shown as the mean ± s.e.m.

	Leak (pyruvate and malate)	OXPHOS; P_CI (D)_ (ADP)	OXPHOS P_CI_ (glutamate)	OXPHOS; P_CI+II_ (succinate)	ETC E_CI+II_ (CCCP)	ETC E_CII_ (rotenone)	ROX (antimycin-A)
Soleus morning-SC^+^	16.6 ±1.8	103.6 ±8.9	120.4 ±10.2	173.1 ±13.3	168.3 ± 12.2	87.8 ±3.8	8.2 ±0.9
Soleus afternoon-SC^+^	16.3 ±1.4	106.2 ± 14.1	121.1 ±16.0	185.1 ±16.4	188.3 ±16.1	95.7 ±7.2	8.1 ±0.6
Soleus evening-SC^+^	12.3 ±2.9	108.4 ±4.2	116.1 ±7.2	147.4 ±12.3	140.2 ±14.2	69.7 ±8.3	8.1 ±0.9
Soleus morning-SC^-^	16.0 ±1.0	102.2 ±7.5	110.2 ±7.4	173.0 ±11.5	185.2 ±19.0	103.7 ±8.7	8.1 ±1.0
Soleus afternoon-SC^-^	16.3 ±2.0	119.1 ±9.8	131.2 ±11.8	183.3 ±9.5	186.6 ±10.0	100.9 ±9.9	9.4 ±1.1
Soleus evening- SC^−^	15.8 ±1.5	111.3 ±4.3	121.9 ±5.5	174.3 ±14.0	171.9 ±15.9	88.6 ±9.3	8.3 ±0.6
TA morning-SC^+^	11.4 ±1.7	71.0 ±9.3	77.2 ±6.4	134.6 ±9.2	139.2 ±9.3	69.7 ±6.8	4.9 ±0.3
TA afternoon-SC^+^	6.9 ±0.6	55.7 ±3.1	67.2 ±2.8	101.5 ±4.8	110.6 ±7.9	60.6 ±2.7	2.9 ±1.5
TA evening-SC^+^	6.8 ±1.2	68.4 ±9.2	80.3 ±11.8	117.3 ±16.3	126.3 ±17.1	68.5 ±7.3	3.0 ±0.7
TA morning-SC^-^	9.2 ±0.6	67.7 ±2.7	81.4 ±4.4	126.5 ±8.0	136.3 ±12.0	68.1 ±5.0	3.6 ±0.4
TA afternoon-SC^-^	7.0 ±1.2	60.2 ±7.1	77.9 ±7.0	113.1 ±10.8	119.2 ±13.6	65.8 ±5.0	3.7 ±0.5
TA evening- SC^−^	6.7 ±1.4	56.6 ±3.9	78.9 ±5.8	110.1 ±7.0	130.5 ±3.6	63.0 ±4.9	3.7 ±1.5

### Mitochondrial respiration in the TA across the time of day in the presence and absence of SCs

**FIGURE 3:**
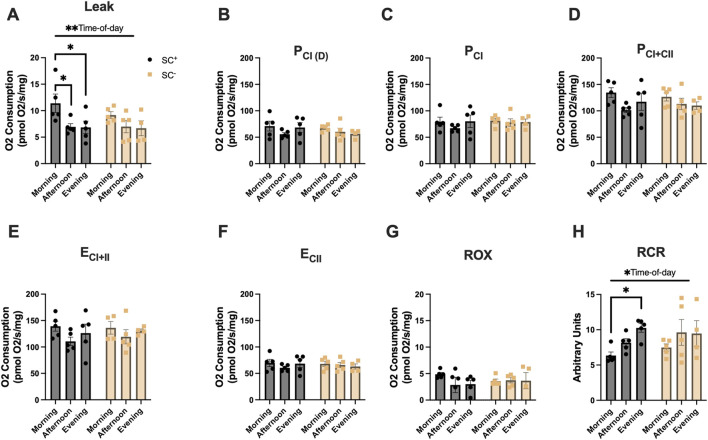
Mitochondrial respiration in the TA across different times of day in the presence and absence of SCs. **(A–H)** Mitochondrial respiration across all states and in the presence/absence of SCs in the TA. All groups were compared using two-way ANOVA for main effects of time-of-day and SC status, followed by Tukey’s post hoc comparisons (n = 4–7 per group) (*p < 0.05). (Leak: PM, P_CI(D): D, P_CI: G, P_CI + II:S, E_CI + II: CCCP, E_CI: R, and ROX: Ama). [P, pyruvate; M, malate; Oct, octanoylcarnitine; D, adenosine diphosphate (ADP); G, glutamate; S, succinate; R, rotenone (inhibitor of complex I); Ama, antimycin A (inhibitor of complex III)].

In the TA (Figures 3A–H), an overall effect of time of day was observed for LEAK-state respiration, and Tukey’s post hoc comparisons showed higher LEAK-state respiration in the morning in SC^+^ animals than at other timepoints, with no such differences observed in SC^−^ groups (Figure 3A; Table 1) (*p* < 0.05). Additionally, an overall effect of time of day was observed for RCR in the TA. Tukey’s post hoc comparisons showed that the RCR was lower in the morning than in the evening in SC^+^ mice, with no differences noted in SC^−^ groups (Figure 3H; Table 1) (*p* < 0.05).

### Citrate synthase activity, complex-I, and mitochondrial DNA copy number across time-of-day and SC groups in glycolytic muscles

**FIGURE 4:**
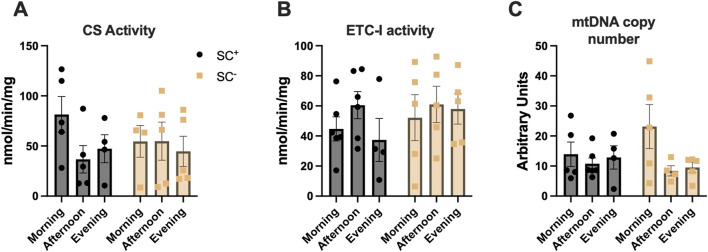
**(A)** Citrate synthase specific activity (nmol/min/mg) **(B)** Complex-I specific activity (nmol/min/mg) and **(C)** Mitochondrial DNA copy number in tibialis anterior muscles across time of day in SC^**+/−**^ groups. All data shown as mean ± s.e.m. All groups compared via two-way ANVOA for main effects of time-of-day and SC status and tukey post-hoc comparisons (n = 4–5 per group).

In the TA muscle, citrate synthase activity, a marker of mitochondrial content, did not differ by the time of day in the presence or absence of SCs (morning-SC^**+**^: 81 ± 18; afternoon-SC^**+**^: 37 ± 14; evening-SC^**+**^: 47 ± 14; morning-SC^**-**^: 55 ± 16; afternoon-SC^**-**^: 55 ± 19; evening-SC^**-**^: 45 ± 15 nmol/min/mg protein) (Figure 4A) (*p* > 0.05). Maximal ETC complex-I activity exhibited no statistical differences across time of day in either SC^+^ or SC^−^ groups (ETC complex-I activity: morning-SC^**+**^: 45 ± 8; afternoon-SC^**+**^: 61 ± 9; evening-SC^**+**^: 37 ± 14; morning-SC^**-**^: 52 ± 15; afternoon-SC^**-**^: 61 ± 12; evening-SC^**-**^: 58 ± 10 nmol/min/mg) (Figure 4B) (*p* > 0.05). The mtDNA copy number demonstrated no differences across time of day in either SC^+^ or SC^−^ groups (mtDNA copy number: morning-SC^**+**^: 14 ± 4; afternoon-SC^**+**^: 11 ± 2; evening-SC^**+**^: 13 ± 4; morning-SC^**-**^: 23 ± 7; afternoon-SC^**-**^: 8 ± 2; evening-SC^**-**^: 10 ± 2; all units reported as mitochondrial copy number) (Figure 4C) (*p* > 0.05).

### Gene expression of muscle molecular clock and mitochondrial genes across time-of-day and SC groups

**FIGURE 5:**
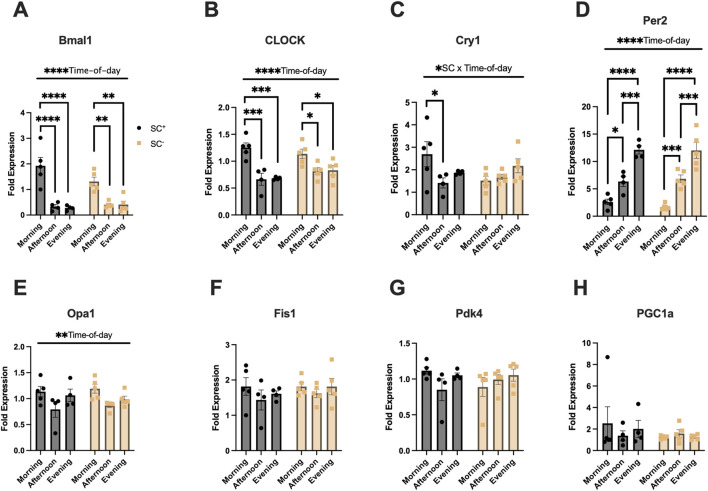
**(A–D)** Muscle molecular clock gene expression (*Bmal1*, *CLOCK*, *Cry1*, *Per2*). **(E–F)** Expression of mitochondrial fusion (*Opa1*) and fission (*Fis1*) genes. **(G–H)** Expression of metabolic genes (*Pdk4* and *Pgc1a*). All gene expression data is of the quadriceps. All data in units of fold-expression. All data shown as mean ± s.e.m. All groups compared via two-way ANVOA for main effects of time-of-day and SC status and tukey post-hoc comparisons (*p < 0.05), (***p < 0.005), (****p < 0.001) (n = 4–5 per group).

Gene expression analysis was performed to determine muscle mRNA levels of specific clock, mitochondrial, and metabolic genes to identify any time-of-day alternations in the presence or following ablation of SCs (Figures 5A–H). *Bmal1*, *CLOCK*, and *Per2* all exhibited significant overall effects for the time of day (*p* < 0.0001) (Figures 5A,B,D). Tukey’s post hoc comparisons showed higher peak expression in *Bmal1* in the morning than in the afternoon/evening in SC^+^ mice (*p* < 0.0001) and SC^−^ mice (*p* < 0.01) (Figure 5A). In *CLOCK* expression, higher expression was observed in the morning than in the afternoon/evening in SC^+^ mice (*p* < 0.001) and SC^−^ mice (*p* < 0.05) (Figure 5B). In *Per2* expression, SC^+^ mice demonstrated the lowest expression compared to afternoon (*p* < 0.05) and evening timepoints (*p* < 0.0001), with similar findings in SC^−^ mice (*p* < 0.001; (*p* < 0.0001) (Figure 5D). Afternoon *Per2* expression was lower than evening expression (*p* < 0.001) in both SC^+^ and SC^−^ animals (Figure 5D). An interaction effect between SC and time of day (*p* < 0.05) was observed for *Cry1* gene expression (Figure 5C). Tukey’s post hoc comparisons revealed that morning *Cry1* expression was higher than that of the afternoon in SC^+^ animals (*p* < 0.05); however, such differences were not observed in SC^−^ animals. Mitochondrial gene *Opa1* exhibited an overall effect of time of day (Figure 5E) (*p* < 0.05), with no significant findings in *Fis1*, *Pdk4*, and *PGC1a* gene expressions.

### Validation of a novel contractile submaximal-fatigue protocol reliant on mitochondria in glycolytic muscles

**FIGURE 6:**
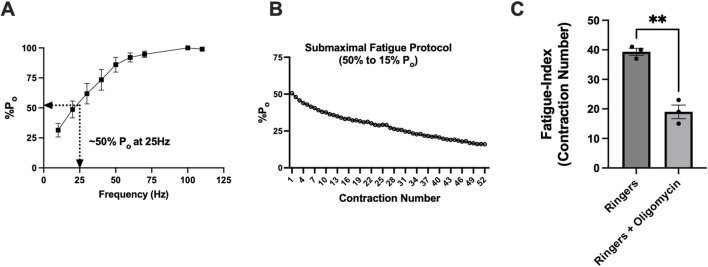
Validation of a novel contractile submaximal-fatigue protocol reliant on mitochondria in glycolytic muscles. **(A)** Force–frequency stimulation curve showing maximal tetanic-specific force (P_o_). The lines indicate that 25 Hz frequency stimulation resulted in 50% P_o_. **(B)** Representative figure showing *ex vivo* fatiguing protocol from 50% P_o_ to 15% P_o_. **(C)** Comparison of the number of contractions until fatigue between control (Ringer’s solution) versus Ringer’s + oligomycin-incubated muscles. The fatigue index indicates the number of contractions until fatigue (defined as 50% P_o_ to 15% P_o_). All data are shown as the mean ± s.e.m. EDL muscles were used for these experiments. All groups were analyzed using unpaired t-tests (**p < 0.01 and ***p < 0.001) (n = 3 per group).

Before assessing whether time-of-day differences in mitochondrial respiration impacted submaximal contractile fatigue (reliant on mitochondrial energy), we first validated that our fatigue protocol relied on mitochondria using oligomycin, a potent inhibitor of mitochondrial ATP synthase. Force–frequency curves were generated on untreated mice to identify the frequency (25 Hz) corresponding to 50% P_o_ (Figure 6A). The number of contractions at 25 Hz required to fatigue muscle from 50% to 15% P_o_ was compared between muscles in Ringer’s solution vs. Ringer’s + oligomycin (representative trace of protocol, Figure 6B). Maximal tetanic force measured immediately before and after 10 min of incubation with oligomycin revealed that force decreased by ∼13% (87% P_o_) (*p* < 0.01). During the fatiguing protocol in the presence of oligomycin, muscle fatigued ∼50% more rapidly than that of control animals, validating the protocol as mitochondria-dependent and inductive of submaximal fatigue (Ringer’s: 39 ± 1 contractions; Ringer’s + oligomycin: 19 ± 2 contractions, *p* < 0.005) (Figure 6C).

### Fatigue-resistance profiles of morning-SC^+^ and afternoon-SC^+^ animals in glycolytic muscles

**FIGURE 7:**
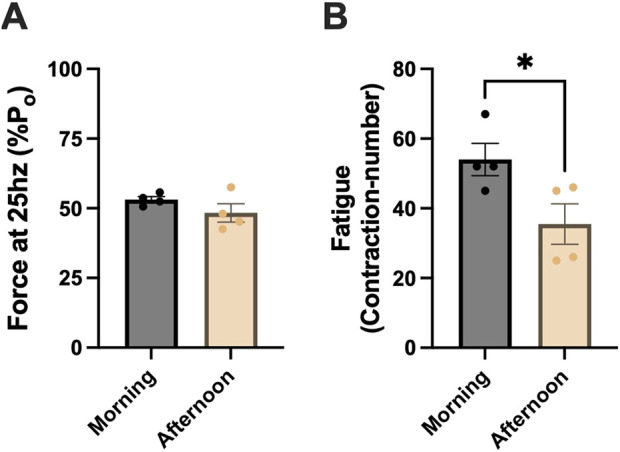
Fatigue-resistance profiles of morning-SC^+^ and afternoon-SC^+^ animals in glycolytic muscle. **(A)** ∼50% of maximal force produced at 25 Hz in morning-SC^**+**^ and afternoon-SC^**+**^ animals. **(B)** Fatigue index in morning-SC^**+**^ and afternoon-SC^**+**^ animals. EDL muscles were used for these experiments. All data are shown as the mean ± s.e.m. All groups were analyzed using unpaired t-tests (*p < 0.05) (n = 3–5 per group).

Next, we assessed whether differences in mitochondrial respiration impacted submaximal contractile fatigue profiles. At 25 Hz, morning-SC+ and afternoon-SC + animals produced ∼50% maximal force (morning-SC^**+**^: 53 ± 1; afternoon-SC^**+**^: 48 ± 3; units in %P_o_) (Figure 7A). During fatiguing contractions, morning-SC^**+**^ animals were more fatigue-resistant, requiring ∼35% more contractions to fatigue than afternoon-SC^**+**^ animals (morning-SC^**+**^: 54 ± 5; afternoon-SC^**+**^: 36 ± 6 contractions) (*p* < 0.05) (Figure 7B).

## Discussion

We report that RCR was the lowest in the SOL and TA, while TA LEAK-state respiration was the highest in the morning, with such time-of-day differences not observed after SC ablation. At the peak and trough timepoints that coincided with *Bmal1* and *CLOCK* molecular clock gene expressions, we measured mitochondria-dependent submaximal fatigue and found that glycolytic muscle (EDL) was ∼35% more fatigue-resistant in the morning than in the afternoon. High LEAK and low RCR in the morning indicate that mitochondrial efficiency may be reduced at this time, and thus, effects on submaximal fatigue are unlikely to result from time-of-day-dependent regulation of mitochondrial energy production. Collectively, our data suggest that the presence of SCs, in part, exerts a time-of-day effect on respiration, but time-of-day effects on mitochondria-dependent fatigue are likely regulated from another circadian node.

As diurnal molecular clocks reside in SCs (Solanas et al., 2017) and SC ablation negatively impacts endurance exercise capacity reliant on mitochondrial energy production (Englund et al., 2020; Jackson et al., 2015), it remains unknown whether SCs exert diurnal influence over mitochondrial function. Several metabolic, contractile, and mitochondrial genes related to exercise performance within SCs display an oscillatory expression profile (Solanas et al., 2017), suggesting that SCs exert time-of-day-dependent downstream regulations on exercise capacity. However, direct assessments of muscle physiology relevant to exercise capacity (i.e., force production and metabolism) across different times of day, in the presence/absence of SCs, are lacking. We recently reported that SC ablation altered maximum isometric- and eccentric-specific force according to time of day (Kahn et al., 2024), adding support to the view that the SC molecular clock’s diurnal transcriptome exerts effects on the corresponding muscle physiology at the functional level. Results from the current study demonstrate that mitochondrial respiration is influenced by time of day in the presence, but not in the absence, of SCs. These results align with our previous work (Kahn et al., 2024; Kahn et al., 2025) and further characterize SC-dependent time-of-day regulation of muscle physiology. Furthermore, in the context of past studies demonstrating altered exercise capacity following SC ablation (Englund et al., 2020; Jackson et al., 2015), one possible mechanism to explain such findings could be related to our observations that SCs play a regulatory role in mitochondrial function.

Although mitochondrial efficiency may be lowest in the morning (as indicated by LEAK and RCR), we observed greater mitochondria-dependent submaximal fatigue resistance at this time. These findings indicate that time-of-day regulations on the contractile kinetics of submaximal fatigue, rather than a direct influence on mitochondria, may underpin differences noted in fatigue profiles. Furthermore, enhanced fatigue resistance in the morning versus afternoon coincided with peak *Bmal1* and *CLOCK* gene expressions, critical regulators of calcium-handling genes during E–C coupling (McCarthy et al., 2007). Although *submaximal* fatigue differed by time of day, others have shown that fatigue induced via *maximal* contractions is not influenced by time of day (Fitzgerald et al., 2024), suggesting that different nodes regulating metabolism may be subjected to divergent time-of-day regulations. In support of this notion, recent work demonstrated that diurnal exercise capacity is dependent on exercise-intensity and this phenomenon is regulated by muscle molecular clocks. Specifically in humans, this work showed that 1 h of submaximal exercise in the morning versus evening displayed no differences in blood lactate accumulation (surrogate of glycolytic flux), whereas participants’ fractional utilization of VO_2_ throughout exercise differed by the time of day (Ezagouri et al., 2019). Our findings of enhanced submaximal fatigue resistance in the morning concur with other studies that reported that both humans and mice display markers of enhanced endurance-capacity during submaximal exercise early in the day (Maier et al., 2021; Ezagouri et al., 2019).

A recent study reported that *ex vivo* fatigue induced by maximal tetanic contractions did not vary with the time of day (Fitzgerald et al., 2024). However, the bioenergetic demands that limit *maximal* versus *submaximal* fatigue are distinct (Hargreaves and Spriet, 2020; Allen et al., 2008), with evidence that control of metabolism may be under separate time-of-day regulation (Ezagouri et al., 2019). In this regard, a previous report demonstrated that inhibiting mitochondria reduced maximal force production; however, rates of fatigue were similar following maximal isometric fatiguing contractions (Zhang et al., 2006). This suggests that mitochondria play a minimal role in *ex vivo* whole-muscle fatigue induced by *maximal* contractions.

The mechanism by which mitochondria-dependent submaximal fatigue differs by time of day may reside at the intersection of mitochondrial energy deliverance to contractile units during submaximal fatigue bouts. This is partially supported by the fact that we observed time-of-day differences in mitochondria-dependent contractile fatigue despite no differences in peak respiration (states 3 and 4). Furthermore, the inverse findings of highest LEAK-state respiration and lowest RCR in the morning indicate that the efficiency of mitochondrial energy production may be compromised in the morning. Low mitochondrial energy production but high fatigue resistance in the morning may suggest that time-of-day effects on submaximal fatigue are unlikely due to regulations on mitochondrial ATP production. Future work should determine whether the kinetics of mitochondrial ATP *deliverance* to sarcomeres is the mechanistic underpinning of time-of-day effects on submaximal fatigue.

Our study has several technical limitations. Our transgenic mouse model (Pax7TDA) that inducibly depletes SCs relies on the Cre-Lox system, which is accomplished via oral gavage of tamoxifen. However, tamoxifen may have unintended metabolic effects on various organ systems, and therefore, tamoxifen treatment is always followed up by a washout period to avoid any unintended effects of tamoxifen. Therefore, in line with past studies, we performed all experimentation following a minimum washout period of 2 weeks (average washout period 26.4 days ±0.97 days SEM) (Kahn et al., 2024; Murach et al., 2021; Englund et al., 2021; Englund et al., 2020; Dayanidhi et al., 2020; Murach et al., 2017; Kinney et al., 2017; Fry et al., 2014; McCarthy et al., 2011; Jackson et al., 2015). Additionally, all experiments were carried out after “lights on,” and therefore, the generalizability of our findings to the active phase may be limited. Animals were fasted for 8–12 h prior to mitochondrial respiration studies. Phenotypes associated with physical activity have been previously described elsewhere in this mouse model (Englund et al., 2020; Jackson et al., 2015). Additionally, previous works have shown that basal physical activity (locomotor, resting energy expenditure, and feeding) measures are circadian in nature (Mansingh et al., 2024; Maier et al., 2021; Kumar et al., 2024), and therefore, mice were not provided with voluntary wheel running and were fasted similarly across groups. Although we applied the necessary controls aligned with prior knowledge of the mouse model and circadian phenotypes, we did not monitor physical activity directly at different times of day, limiting our interpretations of our findings.

## Conclusion

In conclusion, we report that SCs, in part, influence mitochondrial respiration by time of day. Our novel, submaximal, mitochondria-dependent fatigue protocol revealed that fatigue resistance was ∼35% greater in the morning than in the afternoon. Although mitochondrial efficiency (leak and RCR) was lower in the morning, mitochondria-dependent fatigue resistance was higher, suggesting that the underlying mechanism explaining this result may stem from another circadian regulatory node rather than a direct influence on mitochondrial respiration. Collectively, our results suggest that the presence of SCs may affect time-of-day muscle physiology, and future work is warranted to further elucidate the underlying mechanisms.

## Data Availability

The original contributions presented in the study are included in the article/supplementary material, further inquiries can be directed to the corresponding authors.
